# Genomic and transcriptomic characterization reveals B‐cell hyperactivation and immune evasion in hepatitis B virus‐associated diffuse large B‐cell lymphoma

**DOI:** 10.1002/ctm2.1293

**Published:** 2023-06-08

**Authors:** Wei Qin, Nan Wang, Qing Shi, Rui Sun, Zhong Zheng, Di Fu, Lei Dong, Chen Li, Yifang Zhang, Pengpeng Xu, Shu Cheng, Ying Qian, Yan Feng, Li Wang, Weili Zhao

**Affiliations:** ^1^ Shanghai Institute of Hematology State Key Laboratory of Medical Genomics National Research Center for Translational Medicine at Shanghai Ruijin Hospital affiliated to Shanghai Jiao Tong University School of Medicine Shanghai China; ^2^ Department of Pathology Shanghai Ruijin Hospital Shanghai Jiao Tong University School of Medicine Shanghai China; ^3^ Network and Information Center Shanghai Jiao Tong University Shanghai China; ^4^ State Key Laboratory of Microbial Metabolism School of Life Sciences and Biotechnology Shanghai Jiao Tong University Shanghai China; ^5^ Pôle de Recherches Sino‐Français en Science du Vivant et Génomique Laboratory of Molecular Pathology Shanghai China

1

Dear Editor,

Hepatitis B virus (HBV) is an oncogenic virus and a major risk factor for developing hepatocellular carcinoma.^1^ Growing evidence also suggests that HBV infection is linked to an increased incidence of diffuse large B‐cell lymphoma (DLBCL).^1^ Among 1925 newly diagnosed DLBCL patients (Figure S1), we revealed that DLBCLs with current HBV infection (HBV‐surface‐antigen [HBsAg]+), instead of those with previous HBV infection (HBsAg‐ and antibodies against HBV‐core‐antigen+), correlated with high‐risk clinical features (Table S1), as previously reported.^2^, ^3^ The prognostic impact of HBV infection in DLBCL remained controversial in the rituximab era,^2^, ^3^ largely due to the relatively small sample size of patients receiving rituximab‐containing treatment. Here, among 1490 patients with R‐CHOP treatment, we observed that the progression‐free survival (PFS) and overall survival (OS) of DLBCLs with current HBV infection were significantly worse than those of non‐HBV infection (Figure 1A,B). Moreover, current HBV infection independently predicted adverse PFS and OS when other prognostic variables were adjusted (Figure S2). More elderly patients were observed in the previous HBV infection group, probably due to the universal infant HBV vaccination in China since 2002.

**FIGURE 1:**
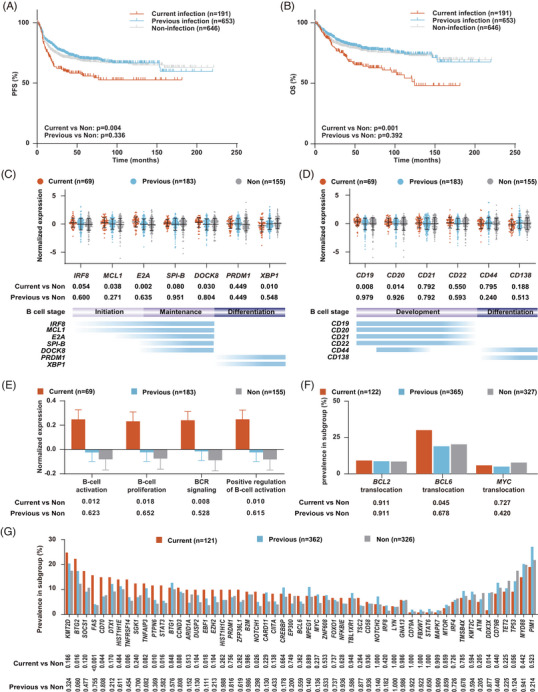
Clinical outcomes, B‐cell status, and genomic alterations of diffuse large B‐cell lymphoma (DLBCL) patients according to hepatitis B virus (HBV) infection. Progression‐free survival (A) and overall survival (B) of DLBCL patients with current, previous, or non‐HBV infection. (C) Normalized expression of transcriptional factors for B‐cell development and differentiation in DLBCL patients with current, previous, or non‐HBV infection. Lower graph shows the dynamic changes of transcriptional factors for different B‐cell stages (germinal center [GC] initiation, GC maintenance, and B‐cell differentiation). (D) Normalized expression of B‐cell surface markers in DLBCL patients with current, previous, or non‐HBV infection. Lower graph shows the dynamic changes of B‐cell surface markers for different B‐cell stages (B‐cell development stage and B‐cell differentiation stage). (E) Enrichment score for B‐cell‐related signaling pathways using single sample Gene Set Enrichment Analysis in DLBCL patients with current, previous, or non‐HBV infection. (F) Prevalence of common chromosomal translocations in DLBCL patients with current, previous, or non‐HBV infection. (G) Prevalence of gene mutations in DLBCL patients with current, previous, or non‐HBV infection.

HBV indirectly promotes DLBCL tumorigenesis by inducing B‐cell hyperactivation.^2^ Accordingly, in DLBCLs of current HBV infection, transcriptional factors for germinal center (GC) initiation and maintenance^4^ were upregulated, while transcriptional factor for B‐cell differentiation was downregulated, as compared to those of non‐HBV infection (Figure 1C). Besides, B‐cell surface markers for B‐cell development^5^ were significantly higher in DLBCLs of current HBV infection than in non‐HBV infection (Figure 1D). Moreover, DLBCLs of current HBV infection presented upregulated B‐cell‐related signaling pathways as revealed by single sample Gene Set Enrichment Analysis (GSEA) (Figure 1E, Table S2), and increased major histocompatibility complex class II molecules (Figure S3A, Table S3), which play an essential role in B cell‐T cell co‐stimulation.^4^ However, no significant difference was detected between previous HBV infection and non‐HBV infection. Upon B‐cell activation, B cells enter into the GC to generate high‐affinity antibodies through activation‐induced cytidine deaminase (AID).^6^ In pathological conditions, enhanced AID activity induces aberrant generation of genetic mutations and chromosomal translocations.^6^ Indeed, DLBCLs with current HBV infection exhibited significantly increased *BCL6* translocation by fluorescence in situ hybridization (Figure 1F), as well as increased gene mutations, including *BTG2*, *FAS*, *CD70*, *TNFRSF14*, *PTPN6*, *STAT3*, *EBF1*, and *NOTCH1* (Figure 1G). However, no significant difference in chromosomal translocations or gene mutations was detected between previous and non‐HBV infection. Moreover, no significant difference of the genetic subtypes (Other, BN2, EZB, MCD, A53, N1, ST2, or genetically composite) was observed among three groups (Figure S3B, Table S4). Together, HBV virus may increase genomic alterations through B‐cell hyperactivation under persistent HBV antigen stimulation, contributing to DLBCL development.

Based on the similar clinical and molecular patterns, patients with previous and non‐HBV infection were grouped as non‐current HBV infection. GSEA revealed that current HBV infection was significantly associated with enriched signaling pathways involving B‐cell activation and immune regulation (Figure 2A), with *CD70* having the highest fold change among the significantly overexpressed genes in current HBV infection group (Figure 2B). By immunohistochemistry, DLBCLs with current HBV infection showed aberrant higher CD70 expression on B‐lymphocytes (Figure 2C), indicating association between B‐cell activation and CD70 expression. By RNA sequencing, enrichment of B‐cell activation positively correlated with *CD70* expression (Figure 2D). To confirm the role of B‐cell activation on *CD70* upregulation, we cultured OCI‐LY10 and SU‐DHL4 cells with anti‐IgG, and observed significantly increased *CD70* expression in both cell lines induced by anti‐IgG (Figure 2E).

**FIGURE 2:**
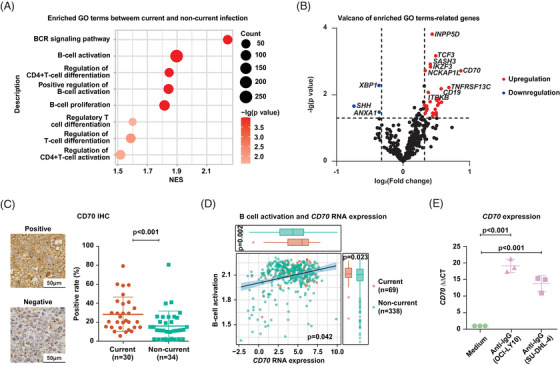
*CD70* upregulation in diffuse large B‐cell lymphoma (DLBCL) patients with current hepatitis B virus (HBV) infection. (A) Upregulated gene oncology terms revealed by Gene Set Enrichment Analysis in DLBCL patients with current HBV infection, as compared to non‐current HBV infection. Size of points indicates number of genes included in each signaling pathway. Color of points indicates −lg (*p* value) of altered signaling pathways between two groups. (B) Volcano plot of differentially expressed genes (DEGs) implicated in the significant signaling pathways between DLBCL patients with current and non‐current HBV infection. Red points indicate significantly upregulated DEGs in DLBCL patients with current HBV infection. Blue points indicate significantly downregulated DEGs in DLBCL patients with current HBV infection. (C) Representative images for CD70 immunohistochemistry (left panel) and percentage of CD70 positivity (right panel) in DLBCL patients with current or non‐current HBV infection. (D) Correlation between B‐cell activation and *CD70* RNA expression in DLBCL patients with current or non‐current HBV infection. (E) *CD70* expression of OCI‐LY10 cells or SU‐DHL4 cells after culturing with anti‐IgG.

T cells play essential roles in chronic HBV infection. As revealed by tumor immunophenotyping, DLBCLs with current HBV infection exhibited significantly increased recruiting activity of regulatory T (Treg) cells (Figure 3A). Treg cells have been recognized to promote immune evasion by controlling the immune activity of T cells.^7^ Indeed, as compared to the Treg‐low group according to the median recruiting score of Treg cells, the Treg‐high group showed significantly downregulated signaling pathways related to T‐cell immune response (Figure S3C). Treg cell increase in patients with current HBV infection was further confirmed by immunohistochemistry (Figure 3B) and multi‐color flow cytometry (Figure 3C, Figure S3D). *CD27*‐*CD70* interaction induces critical survival factors for Treg cells, thereby promoting Treg cell differentiation and inhibiting Treg cell apoptosis.^8^ Consistently, elevated *CD70* expression (*CD70*‐high group based on the median *CD70* level) was significantly related to higher recruiting activity of Treg cells (Figure S3E). Additionally, key chemokines and chemokine receptors involved in Treg recruitment activity were significantly upregulated in *CD70*‐high group, as compared to *CD70*‐low group (Figure S3F). With ectopic expression of *CD70* through transfecting with *CD70*‐vector in OCI‐LY10 and SU‐DHL4 cells (Figure 3D,E), *CD70* facilitated lymphoma cell proliferation in both cell lines (Figure 3F), in consistent with previous study that *CD70* may induce a growth advantage of B cells.^9^ Under co‐culture system with peripheral blood mononuclear cells, CD70 overexpression on OCI‐LY10 and SU‐DHL4 cells significantly promoted Treg cell increasing (Figure 3G) and tumor cell proliferation as well (Figure 3H). Therefore, HBV infection may increase Treg cells via CD70, thereby inducing tumor escape from immune surveillance and subsequent DLBCL progression.

**FIGURE 3:**
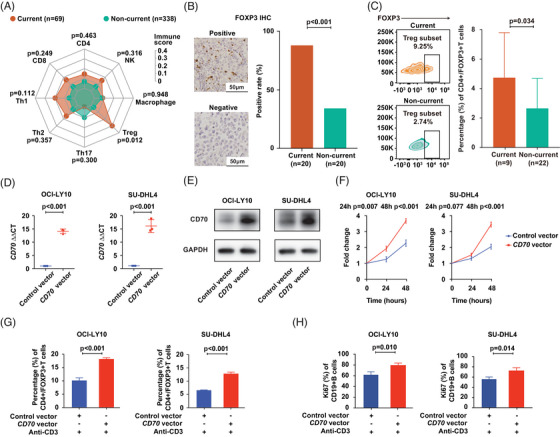
*CD70* induced tumor microenvironmental alterations in diffuse large B‐cell lymphoma (DLBCL) patients with current hepatitis B virus (HBV) infection. (A) Recruiting activity scores for immune cells of the tumor microenvironment in DLBCL patients with current or non‐current HBV infection. (B) Representative images for FOXP3 immunohistochemistry (left panel) and percentage of FOXP3 positivity (right panel) in DLBCL patients with current or non‐current HBV infection. (C) Percentage of regulatory T (Treg) cells in DLBCL patients with current or non‐current HBV infection assessed by multi‐color flow cytometry. Representative contour maps for current or non‐current HBV infection are indicated at the left. (D) *CD70* expression of OCI‐LY10 cells (left panel) or SU‐DHL4 cells (right panel) transfected with *CD70* vector or control vector. (E) Protein expression of CD70 detected in OCI‐LY10 cells (left panel) or SU‐DHL4 cells (right panel) transfected with *CD70* vector or control vector by western blot. GAPDH was used as the loading control. (F) Cell proliferation of OCI‐LY10 cells (left panel) or SU‐DHL4 cells (right panel) transfected with *CD70* vector or control vector. (G) Percentage of Treg cells in peripheral blood mononuclear cells co‐cultured with OCI‐LY10 cells (left panel) or SU‐DHL4 cells (right panel) transfected with *CD70* vector or control vector. (H) Ki‐67 positivity of CD19+B cells in the co‐culture systems of OCI‐LY10 cells (left panel) or SU‐DHL4 cells (right panel) transfected with *CD70* vector or control vector. All the error bars denote standard deviation of three experiments.

Lenalidomide is an effective immunomodulatory agent in treating DLBCL and exerts anti‐tumor activity through targeting Treg cells.^10^ Upon lenalidomide treatment, both co‐culture systems elicited significantly decreased Treg cells (Figure 4A) and tumor cells (Figure 4B). We next treated 52 relapsed/refractory patients with rituximab, ifosfamide, carboplatin, and etoposide (R‐ICE), and observed DLBCLs with current HBV infection displayed significantly shorter PFS and OS than those of non‐current HBV infection (Figure 4C). When relapsed/refractory patients received lenalidomide plus R‐ICE, lenalidomide significantly improved the PFS and OS of DLBCLs with current HBV infection (Figure 4D). Of note, Treg cells remained unchanged upon R‐ICE treatment (Figure 4E), but were significantly decreased upon lenalidomide plus R‐ICE treatment in patients with current HBV infection (Figure 4F).

**FIGURE 4:**
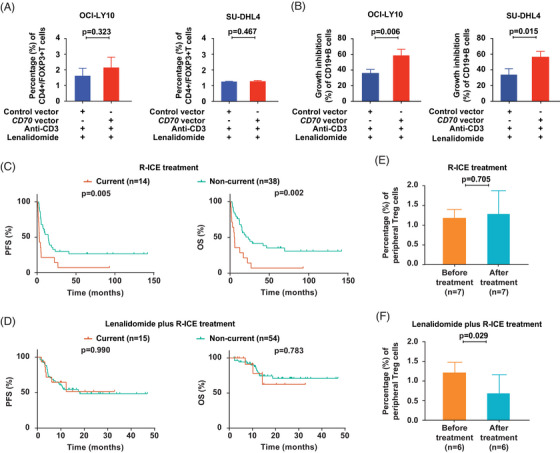
Lenalidomide reversed CD70‐mediated regulatory T (Treg) immunosuppression in diffuse large B‐cell lymphoma (DLBCL) patients with current hepatitis B virus (HBV) infection. (A) Percentage of Treg cells in peripheral blood mononuclear cells co‐cultured with OCI‐LY10 cells (left panel) or SU‐DHL4 cells (right panel) transfected with *CD70* vector or control vector in medium treated with lenalidomide. (B) Growth inhibition of CD19+B cells in the co‐culture systems of OCI‐LY10 cells (left panel) or SU‐DHL4 cells (right panel) transfected with *CD70* vector or control vector in medium treated with lenalidomide. All the error bars denote standard deviation of three experiments. (C) Progression‐free survival (PFS, left panel) and overall survival (OS, right panel) of relapsed or refractory DLBCL patients with current or non‐current HBV infection receiving R‐ICE (rituximab, ifosfamide, carboplatin, and etoposide) treatment. (D) PFS (left panel) and OS (right panel) of relapsed or refractory DLBCL patients with current or non‐current HBV infection receiving lenalidomide plus R‐ICE treatment. (E) Peripheral Treg cells of relapsed or refractory DLBCL patients with current HBV infection before or after R‐ICE treatment. (F) Peripheral Treg cells of relapsed or refractory DLBCL patients with current HBV infection before or after lenalidomide plus R‐ICE treatment.

In conclusion, DLBCLs with current HBV infection may refer as a specific entity with aggressive disease course and resistance to immunochemotherapy. Targeting tumor microenvironment could be promising approaches for mechanism‐based treatment on virus‐related B‐cell malignancies.

## CONFLICT OF INTEREST STATEMENT

The authors declare that they have no conflict of interest.

## Supporting information

Supplementary Information

Supplementary Information
