# Supplementary material for: Genomic and transcriptomic characterization reveals B‐cell hyperactivation and immune evasion in hepatitis B virus‐associated diffuse large B‐cell lymphoma
Source: Clin Transl Med. 2023 Jun 8;13(6):e1293. doi: 10.1002/ctm2.1293 (PMC10248824; doi:10.1002/ctm2.1293)
Supplement: Supplementary file 1 — Supplementary Information [file CTM2-13-e1293-s002.docx]

**Genomic and transcriptomic characterization reveals B-cell hyperactivation and immune evasion in hepatitis B virus-associated diffuse large B-cell lymphoma**

**Running title:** Molecular profiling in HBV-associated DLBCL

Wei Qin,^1,*^ Nan Wang,^1,*^ Qing Shi,^1,*^ Rui Sun,^1,*^ Zhong Zheng,^1^ Di Fu,^1^ Lei Dong,^2^ Hongmei Yi,^2^ Chen Li, ^3^ Yifang Zhang, ^3^ Pengpeng Xu,^1^ Shu Cheng,^1^ Ying Qian,^1^ Yan Feng,^4^ Li Wang,^1,5^ Weili Zhao^1,5^^#^

^1^ Shanghai Institute of Hematology, State Key Laboratory of Medical Genomics, National Research Center for Translational Medicine at Shanghai, Ruijin Hospital affiliated to Shanghai Jiao Tong University School of Medicine, Shanghai, China;

^2^ Department of Pathology, Shanghai Ruijin Hospital, Shanghai Jiao Tong University School of Medicine, Shanghai, China;

^3^ Network and Information Center, Shanghai Jiao Tong University, Shanghai, China;

^4^ State Key Laboratory of Microbial Metabolism, School of Life Sciences and Biotechnology, Shanghai Jiao Tong University, Shanghai, China;

^5^ Pôle de Recherches Sino-Français en Science du Vivant et Génomique, Laboratory of Molecular Pathology, Shanghai, China.

^*^ These authors contributed equally to this study.

**Supplementary Materials and Methods**

**Patients**

A flowchart describing the patient selection is outlined in Figure S1. Excluding 48 patients with primary central nervous system lymphoma, 55 patients with primary mediastinal large B-cell lymphoma, or 2 patients with hepatic C virus infection, a total of 1925 from 2030 newly diagnosed diffuse large B-cell lymphoma (DLBCL) patients with available serological hepatitis B virus (HBV) markers were enrolled in this study. Histological diagnosis was reviewed according to 2016 WHO classification of lymphoid neoplasms.^1^ Clinical characteristics including age, Eastern Cooperative Oncology Group performance status, Ann Arbor stage, serum lactate dehydrogenase, extranodal involvement, liver or spleen involvement, and International Prognostic Index were collected. Excluding 435 patients receiving treatment other than rituximab, cyclophosphamide, doxorubicin, vincristine, and prednisone (R-CHOP) regimen, 1490 patients were included in survival analysis. Tumor samples of 809 patients were analyzed for genetic aberrations using whole-genome sequencing (WGS, n=113), whole-exome sequencing (WES, n=205) or targeted sequencing (n=491). RNA sequencing was performed on 407 patients with qualified tumor samples to explore gene expression pattern and tumor immunophenotyping (TIP). The study was approved by the Shanghai Ruijin Hospital Review Board, and informed consent was obtained in accordance with the Declaration of Helsinki.

**HBV marker examination**

Baseline serological HBV markers were examined using Abbott Alinity System, including HBV surface-antigen (HBsAg), antibodies against HBV surface-antigen (anti-HBs), antibodies against HBV core-antigen (anti-HBc), HBV envelope-antigen (HBeAg), and antibodies against HBV envelope-antigen (anti-HBe). HBV infection status was defined based on these serological markers, including current HBV infection (HBsAg+, n=297), previous HBV infection (HBsAg- and anti-HBc+, n=838), and non-HBV infection (HBsAg- and anti-HBc-, n=790).^2^

**Fluorescence in situ hybridization (FISH) and immunohistochemistry (IHC)**

Tumor samples of 814 patients were analyzed for *BCL2*, *BCL6*, and *MYC* translocations using FISH, as previously described,^3^ including 311 patients with WGS/WES data. *BCL2*/*IGH* fusion probe (Cat# F01008-01, Beijing Jinpujia), *BCL6* break apart probe (Cat# F01008-01, Beijing Jinpujia), and *MYC* break apart probe (Cat# F01008-01, Beijing Jinpujia) were applied to detect *BCL2*, *BCL6*, or *MYC* translocation. Signals were counted in 200 cells and considered positive when > 10% of the tumor cells exhibited a fusion signal for *BCL2*, or a break apart signal for *BCL6* or *MYC*. IHC of available tumor samples was performed on 5 μm paraffin sections using antibodies against BCL6 (Cat# PG-B6p, DAKO), CD10 (Cat# 56C6, DAKO), MUM1 (Cat# MUM1p, DAKO), BCL2 (Cat# 124, DAKO), MYC (Cat# ZA-0555, Beijing Zhongshan), FOXP3 (Cat# ab20034, Abcam), and CD70 (Cat# ab133398, Abcam). Of them, IHC of BCL6, CD10, and MUM1 were carried out on 1552 cases (including 231 current HBV infection, 690 previous HBV infection, and 631 non-HBV infection). Hans algorithm was used to determine germinal center B-cell (GCB) or non-GCB origin, with 30% cut-off values of BCL6, CD10, and MUM1.^4^ BCL2 and MYC staining were performed on 1197 patients (including 174 current HBV infection, 531 previous HBV infection, and 492 non-HBV infection) , with cut-off values defined as 50% and 40%, respectively.^1^ IHC of FOXP3 was performed on 40 cases (including 20 current HBV infection and 20 non-current HBV infection) and staining > 0% was referred as positive.^5^ CD70 staining was applied in 64 cases (including 30 current HBV infection and 34 non-current HBV infection). The percentage of positive cells was calculated and the average of five fields was used to evaluate CD70 positive rate.^6^

**DNA sequencing**

DNA sequencing was performed on 809 patients, including WGS (n=113), WES (n=205), and targeted sequencing (n=491). Among them, 88 WGS and 204 WES were reported in our previous studies,^7-9^ and the remaining data were newly added. WGS was carried out on frozen tumor tissue, and WES on frozen tumor tissue or formalin-fixed paraffin-embedded (FFPE) tumor tissue with quality controlled by agarose gel electrophoresis. Targeted sequencing of 55 lymphoma-associated genes was performed on FFPE tumor tissue. Detailed procedures for DNA sequencing were carried out as previous reported.^7^

**Genetic subtypes**

For 311 patients with available WGS/WES and FISH data, the LymphGen probabilistic classification tool (R code version, https://doi.org/10.5281/zenodo.3700087) was employed to identify genetic subtypes of DLBCL.^10^ Patients were classified into eight genetic subtypes (Other, BN2, EZB, MCD, A53, N1, ST2, and genetically composite) according to the integrated analyses of fusions, gene mutations, and copy-number alterations.

**RNA sequencing**

RNA sequencing was performed on 407 of 809 patients with DNA sequencing data, including 312 patients from our previous studies,^7-9^ and 95 patients newly added. Read pairs were aligned to Ref-seq hg19 by Burrows-Wheeler Aligner version 0.7.13-r1126. Transcript counts table files were obtained using the HTSeq.^11^ R package “limma” was applied to normalize the raw reads and generate differentially expressed genes (DEGs). DEGs with the fold change > 1.25 or < -1.25 and the *p* value < 0.05 were considered significant. Detailed procedures for RNA sequencing were performed as previous reported.^7^

**Gene Set Enrichment Analysis (GSEA)**

Gene expression pattern analysis was performed using GSEA (v4.1.0) by overlapping the genes in a module with the Gene Ontology gene sets of MSigDB (Molecular Signatures Database).^12,13^ Gene markers were ranked using Signal2Noise to generate the gene list. Analysis was run on 1000 permutations by default to evaluate the statistical significance of the enrichment score for each gene sets. Gene sets with the *p* value < 0.05 and the false discovery rate (FDR) < 0.25 were considered statistically significant. Enrichment scores of specific gene sets for each patient were calculated using single sample GSEA (ssGSEA).^14^

**TIP**

The tumor microenvironment was evaluated by TIP (http://biocc.hrbmu.edu.cn/TIP/).^15^ A total of 178 signature genes were grouped into 23 signature sets to characterize the immune activity of the cancer-immunity cycle and the recruitment activity of specific immune cells. The activity score for each signature set was measured based on the RNA sequencing data of individual samples, with the stimulatory and inhibitory genes calculated separately. The final activity score was produced by calculating the difference between the normalized scores of stimulatory gene sets and inhibitory gene sets.

**Cell lines and reagents**

B-cell lymphoma cell lines OCI-LY10 (kindly provided by Huang CX) was grown in IMDM (Cat# 12440-053, Gibco), and SU-DHL4 (available from American Type Culture Collection, Manassas, VA, USA) in RPMI-1640 medium (Cat# 11875119, Gibco), supplemented with 10% heat-inactivated fetal bovine serum (Cat# 10100147, Gibco) and 1% penicillin/streptomycin (Cat# 15140122, Gibco) in a humidified atmosphere (95% air and 5% CO_2_) at 37°C. Immunomodulatory agent lenalidomide (Cat# A2005) was obtained from Selleck Chemicals (Houston, TX, USA).

**Cell transfection**

Lentiviral particles containing purified plasmids *CD70* and vector were transfected into OCI-LY10 and SU-DHL4 cell lines by Lipofectamine 3000 transfection reagents (Cat# L3000015, Invitrogen). The stably transfected clones were selected by green fluorescence protein using BD FacsAria. Cell viability was examined by CCK8 (Cat# CK04, Dojindo) and the absorbance was assessed at 450 nm by spectrophotometry. OCI-LY10 and SU-DHL4 cells were cultured with anti-IgG (Cat# 397302, Biolegend) to confirm the role of B-cell activation on *CD70* upregulation.^16^

***In vitro* co-culture system**

Peripheral blood mononuclear cells (PBMCs) were isolated from peripheral blood of healthy volunteers by Ficoll using density gradient centrifugation, as previously reported.^17^ OCI-LY10 and SU-DHL4 cells (2×10^5^ cells/ml), as well as PMBCs (2×10^6^ cells/ml), were co-cultured in complete α-MEM medium (Cat# 12571063, Gibco) for 24 hours.

**Quantitative real-time PCR**

Total RNA was extracted by TRIzol reagent (Cat# 15596026, Invitrogen) and complementary DNA was synthesized using PrimeScript RT Reagent Kit with gDNA Eraser (Cat# RR047A, TaKaRa). Quantitative real-time PCR was carried out using SYBR Premix Ex TaqTM II (Cat# RR820A, TaKaRa) and ABI ViiA 7 (Applied Biosystems) with following primers: *CD70* (Forward: 5’-GCTTTGGTCCCATTGGTCG-3’, Reverse: 5’-CGTCCCACCCAAGTGACTC-3’), and *GAPDH* (Forward: 5’-CAGGAGGCATTGCTGATGAT-3’, Reverse: 5’-GAAGGCTGGGGCTCATTT-3’) as an endogenous control.

**Western blot**

Protein lysates were obtained using 200 μl lysis buffer (0.5 M Tris-HCl, pH 6.8, 2 mM EDTA, 10% glycerol, 2% SDS, and 5% β-mercaptoethanol), and were electrophoresed on 10% SDS polyacrylamide gels and transferred to nitrocellulose membranes. After blocking with 5% non-fat dried milk, membranes were incubated overnight at 4°C with the primary antibody against CD70 (Cat# 72094, Cell Signaling Technologies) and a horseradish peroxidase-conjugated goat anti-rabbit IgG (Cat# 98164S, Cell Signaling Technologies). GAPDH (Cat# 5174S, Cell Signaling Technologies) was applied to confirm equivalent protein loading. Immunocomplexes visualization was performed using a chemiluminescence phototope-horseradish peroxidase Kit (Cat# 12630S, Cell Signaling Technologies). The intensities of the immunoreactive band were measured using ImageJ.

**Multi-color flow cytometry**

To measure the percentage of immune cells, multi-color flow cytometry was performed on primary tumor samples (9 patients with current HBV infection and 22 patients with non-current HBV infection) and co-culture system cells. Cells were stained with commercial antibodies including Fixable Viability Stain 440UV (Cat# 566332, BD Pharmingen), anti-CD45 (Cat# 563792, BD Pharmingen), anti-CD19 (Cat# 555415, BD Pharmingen), anti-CD3 (Cat# 612940, BD Pharmingen), anti-CD4 (Cat# 624298, BD Pharmingen), anti-CD8 (Cat# 563919, BD Pharmingen), anti-T-bet (Cat# 624295, BD Pharmingen), anti-GATA3 (Cat# 560405, BD Pharmingen), anti-RORγt (Cat# 624290, BD Pharmingen), anti-FOXP3 (Cat# 53-4776-42, BD Pharmingen), anti-CD11b (Cat# 562632, BD Pharmingen), and anti-CD68 (Cat# 624380, BD Pharmingen) antibodies. The cells were collected using BD FACSymphony A5. Data were analyzed by Kaluza C software (Beckman Coulter, Brea, CA, USA).

**Statistical analysis**

Baseline characteristics of patients were compared by Pearson’s χ2 test or Fisher’s exact test. Differences of normalized gene expression and immune activity scores between groups were assessed by *t*-test. *In vitro* experimental data from three separate experiments were analyzed by *t*-test and indicated by mean±standard deviation. Progression-free survival was calculated from the diagnosis date to the date of disease progression or the date of last follow-up. Overall survival was measured from the diagnosis date to the date of death or the date of last follow-up. Survival analysis was generated using the Kaplan-Meier method and compared by the log-rank test. Univariate hazard estimates were generated with the Cox regression methods. Variables with *p* value < 0.10 on univariate analyses were included in the multivariate sets. All *p* values of multiple comparisons were adjusted using the FDR method.^18^ Statistical significance was defined as *p* value < 0.05. All statistical analyses were performed using Statistical Package for the Social Sciences (SPSS) 22.0 software (SPSS Inc., Chicago, USA).

**References**

1. Swerdlow SH, Campo E, Pileri SA, et al. The 2016 revision of the World Health Organization classification of lymphoid neoplasms. *Blood*. 2016;127(20):2375-2390.

2. Lok AS, Everhart JE, Di Bisceglie AM, Kim HY, Hussain M, Morgan TR. Occult and previous hepatitis B virus infection are not associated with hepatocellular carcinoma in United States patients with chronic hepatitis C. *Hepatology*. 2011;54(2):434-442.

3. Ventura RA, Martin-Subero JI, Jones M, et al. FISH analysis for the detection of lymphoma-associated chromosomal abnormalities in routine paraffin-embedded tissue. *J Mol Diagn*. 2006;8(2):141-151.

4. Hans CP, Weisenburger DD, Greiner TC, et al. Confirmation of the molecular classification of diffuse large B-cell lymphoma by immunohistochemistry using a tissue microarray. *Blood*. 2004;103(1):275-282.

5. Guo XY, Zhang GH, Wang ZN, et al. A novel Foxp3-related immune prognostic signature for glioblastoma multiforme based on immunogenomic profiling. *Aging (Albany NY)*. 2021;13(3):3501-3517.

6. Balsas P, Veloza L, Clot G, et al. SOX11, CD70, and Treg cells configure the tumor-immune microenvironment of aggressive mantle cell lymphoma. *Blood*. 2021;138(22):2202-2215.

7. Shen R, Xu PP, Wang N, et al. Influence of oncogenic mutations and tumor microenvironment alterations on extranodal invasion in diffuse large B-cell lymphoma. *Clin Transl Med*. 2020;10(7):e221.

8. Qin W, Fu D, Shi Q, et al. Molecular Heterogeneity in Localized Diffuse Large B-Cell Lymphoma. *Front Oncol*. 2021;11:638757.

9. Huang YH, Cai K, Xu PP, et al. CREBBP/EP300 mutations promoted tumor progression in diffuse large B-cell lymphoma through altering tumor-associated macrophage polarization via FBXW7-NOTCH-CCL2/CSF1 axis. *Signal Transduct Target Ther*. 2021;6(1):10.

10. Wright GW, Huang DW, Phelan JD, et al. A Probabilistic Classification Tool for Genetic Subtypes of Diffuse Large B Cell Lymphoma with Therapeutic Implications. *Cancer Cell*. 2020;37(4):551-568.e514.

11. Anders S, Pyl PT, Huber W. HTSeq--a Python framework to work with high-throughput sequencing data. *Bioinformatics*. 2015;31(2):166-169.

12. Mootha VK, Lindgren CM, Eriksson KF, et al. PGC-1alpha-responsive genes involved in oxidative phosphorylation are coordinately downregulated in human diabetes. *Nat Genet*. 2003;34(3):267-273.

13. Subramanian A, Tamayo P, Mootha VK, et al. Gene set enrichment analysis: a knowledge-based approach for interpreting genome-wide expression profiles. *Proc Natl Acad Sci U S A*. 2005;102(43):15545-15550.

14. Hänzelmann S, Castelo R, Guinney J. GSVA: gene set variation analysis for microarray and RNA-seq data. *BMC Bioinformatics*. 2013;14:7.

15. Xu L, Deng C, Pang B, et al. TIP: A Web Server for Resolving Tumor Immunophenotype Profiling. *Cancer Res*. 2018;78(23):6575-6580.

16. Dang LV, Nilsson A, Ingelman-Sundberg H, et al. Soluble CD27 induces IgG production through activation of antigen-primed B cells. *J Intern Med*. 2012;271(3):282-293.

17. Zheng Z, Sun R, Zhao HJ, et al. MiR155 sensitized B-lymphoma cells to anti-PD-L1 antibody via PD-1/PD-L1-mediated lymphoma cell interaction with CD8+T cells. *Mol Cancer*. 2019;18(1):54.

18. Benjamini Y, Hochberg Y. Controlling the False Discovery Rate: A Practical and Powerful Approach to Multiple Testing. *Journal of the Royal Statistical Society Series B (Methodological)*. 1995;57(1):289-300.

**Supplementary Tables**

**Supplementary Table S1. Clinical and pathological characteristics of DLBCL patients according to HBV infection status**

**Supplementary Table S2. Gene signatures for B-cell-related signaling pathways**

**Supplementary Table S3. Gene signatures for MHC class I and class II molecules**

**Supplementary Table S4. Molecular subtypes and mutations of 55 lymphoma-associated genes in DLBCL patients**

**Supplementary Figures**

**Supplementary Figure S1**

**
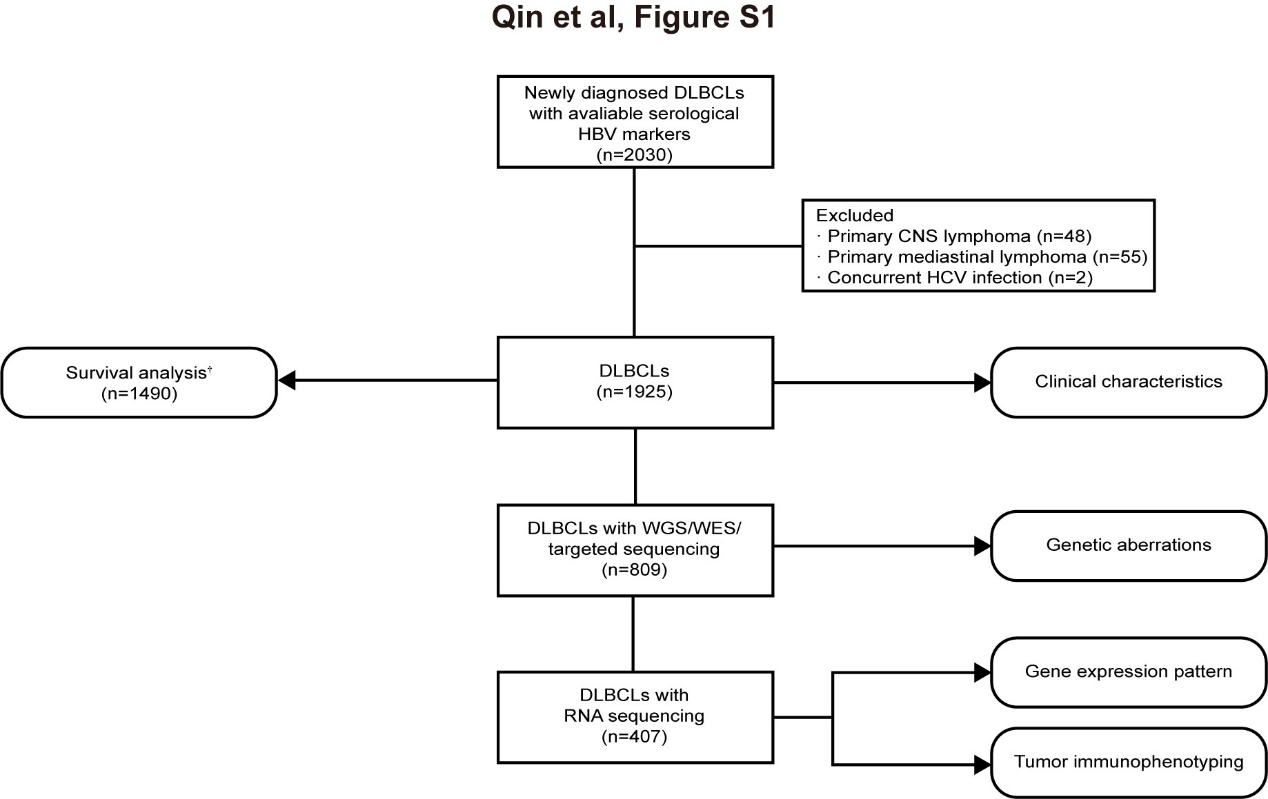
**

^†^ Excluded 435 diffuse large B-cell lymphoma patients receiving treatment other than rituximab, cyclophosphamide, doxorubicin, vincristine, and prednisone (R-CHOP) regimen. Abbreviations: HBV, hepatitis B virus; CNS, central nervous system; HCV, hepatitis C virus; WGS, whole-genome sequencing; WES, whole-exome sequencing.

**Supplementary Figure S2**

**
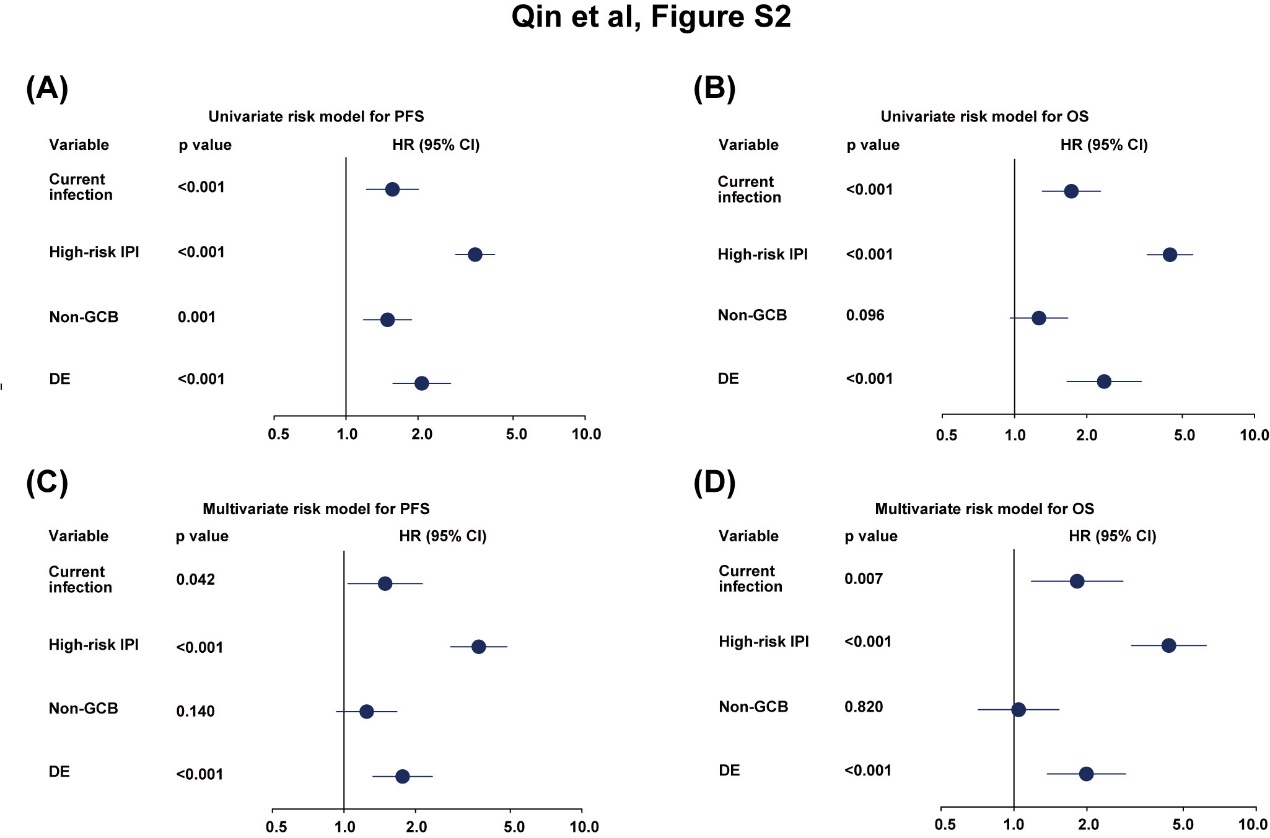
**

(A-B) Univariate risk models of predictors indicated on the left (current Hepatitis B virus infection, high-risk International Prognostic Index, non-germinal center B-cell subtype, and BCL2/MYC double expressors) for progression-free survival (PFS, A) and overall survival (OS, B) in diffuse large B-cell lymphoma (DLBCL). (C-D) Multivariate risk models for PFS (C) and OS (D) in DLBCL using predictors with *p* value < 0.10 on univariate risk models.

**Supplementary Figure S3**

**
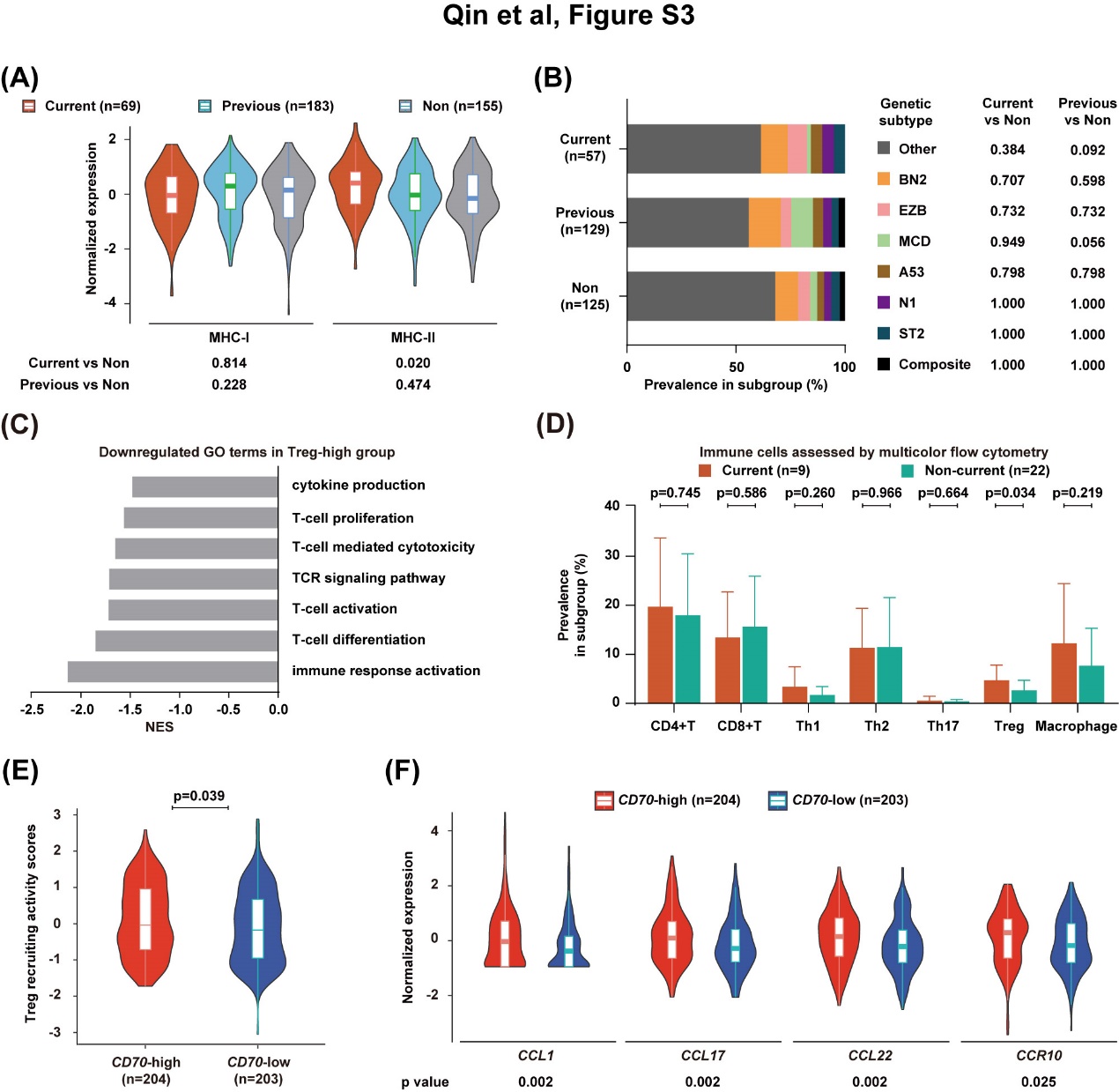
**

(A) Normalized expression of major histocompatibility complex molecules in diffuse large B-cell lymphoma (DLBCL) patients with current, previous, or non-hepatitis B virus (HBV) infection. (B) Prevalence of genetic subtypes in DLBCL patients with current, previous, or non-HBV infection. (C) Downregulated gene oncology terms revealed by Gene Set Enrichment Analysis in the regulatory T cell (Treg)-high group, as compared to the Treg-low group. (D) Prevalence of immune cells in DLBCL patients with current or non-current HBV infection assessed by multi-color flow cytometry, including CD4+T cells, CD8+T cells, Th1, Th2, Th17, Treg cells, and macrophages. (E) Recruiting activity score of Treg cells in the *CD70*-high or *CD70*-low groups. (F) Normalized expression of chemokines and chemokine receptors involved in the Treg recruiting activity of the *CD70*-high or *CD70*-low groups.
